# Association between dietary habits and emotional and behavioral problems in children: the mediating role of self-concept

**DOI:** 10.3389/fnut.2025.1426485

**Published:** 2025-03-07

**Authors:** Dong Zhao, Wenhan Xiao, Boren Tan, Ye Zeng, Shuting Li, Jiali Zhou, Shiyi Shan, Jing Wu, Qian Yi, Ronghua Zhang, Danting Su, Peige Song

**Affiliations:** ^1^Department of Nutrition and Food Safety, Zhejiang Provincial Center for Disease Control and Prevention, Hangzhou, Zhejiang, China; ^2^Center for Clinical Big Data and Statistics of the Second Affiliated Hospital Zhejiang University School of Medicine, School of Public Health, Zhejiang University School of Medicine, Hangzhou, China; ^3^Department of Sociology, Zhejiang University, Hangzhou, Zhejiang, China; ^4^Human Cognitive Neuroscience, Department of Psychology, University of Edinburgh, Edinburgh, United Kingdom

**Keywords:** emotional problems, behavioral problems, self-concept, dietary habits, diet

## Abstract

**Introduction:**

Increasing research has focused on the influence of diet on mental health and well-being. This study aimed to investigate dietary habits status and their associations with emotional and behavioral problems (EBPs) in pre-teen children, as well as explore the mediating effect of child self-concept in the associations between healthy dietary habits and EBPs.

**Methods:**

A cross-sectional survey using stratified random sampling was conducted to recruit third-grade children and their caregivers. Dietary habits and self-concept were assessed with self-administrated questionnaires in children. Information on children’s EBPs was collected through questionnaires completed by their caregivers. Multilevel logistic regression models were used to estimate the associations between dietary habits and self-concept and EBPs, respectively. The mediation analysis was employed to test the mediating role of self-concept in the association between dietary habits and EBPs.

**Results:**

Of 1,126 caregiver-child dyads (Mean age of children: 9.53, 52.8% boys) included, only 37.4 and 54.2% of children met the healthy standard of milk/soy milk and fruit, respectively. Healthy fresh fruit (odds ratio [OR] = 0.57, 95% confidence intervals [CI] 0.40–0.78) and vegetables intake (OR = 0.54, 95% CI 0.38–0.76) were associated with a higher self-concept while frequent consumption of sweet foods (OR = 1.58, 95% CI 1.05–2.36) and street foods (OR = 1.61, 95% CI 1.14–2.28) were associated with a lower self-concept. Children who had unhealthy sugar-sweetened beverages intake were at an elevated risk of EBPs (OR = 1.41, 95% CI 1.03–1.95). Moreover, the relationship between healthy dietary habits and EBPs was mediated by self-concept (indirect effect *β* = −0.09, *p* < 0.001, total effect *β* = −0.13, *p* < 0.001), the proportion of mediation was 29%.

**Conclusion:**

This study revealed that the dietary habits of pre-adolescents need improvement, and dietary habits of certain foods, such as fresh fruits and sugar-sweetened beverages, were significantly associated with child mental health. Furthermore, dietary practices were related to the reduced EBPs through an enhanced self-concept. The findings provide an evidence base for developing dietary improvement strategies for pre-adolescent children in families, schools, and other health service settings, thereby contributing to the United Nations’ Sustainable Development Goals related to zero hunger and good health and well-being.

## 1 Introduction

Emotional and behavioral problems (EBPs) are two main dimensions of mental disorders (1). The Global Burden of Disease Study 2019 found that EBPs affected 12.4% of children aged 10–14 years globally (2). In Asian children, EBPs were also notably prevalent, with an overall rate of 11.8% varied by different regions (3). In China specifically, diagnosis rates also reached 3.0% for depression disorders, 4.7% for anxiety disorders and 10.2% for attention-deficit and disruptive disorders (ADHD) in 2022, underscoring the need for public health attention (4). Moreover, EBPs are significantly associated with social rejection (5), academic failure (6), and suicidal ideation (7) in children. Given the significant disease burden, it is crucial to investigate the risk factors and underlying mechanism of EBPs for targeted management.

The United Nations’ Sustainable Development Goals of Zero Hunger (SDG 2) and Good Health and Well-being (SDG 3) place a significant emphasis on maintaining nutritious diets (8). In line with this global agenda, promoting healthy dietary habits is critical to ensure nutrition and energy for the optimal childhood health, including a reduction in EBPs (9). An increasing number of studies have claimed the potential mechanism between dietary habits and EBPs through the gut-brain connection system, mediated by neurotransmitters, neuropeptides, and inflammation (10). For example, pro-inflammatory diets, characterized in high in processed foods, sugars, have been found associated with chronic inflammation in the brain (11). This sustained inflammation is thought to contribute to diverse mental health problems, such as depression and anxiety (12).

Empirical studies have also revealed the association between overall healthy diet and EBPs among children. For instance, a meta-analysis based on 14 observational studies revealed that healthy dietary patterns were associated with a 35% lower incidence of ADHD in children (13). However, the evidence regarding associations between certain foods and child EBPs remains limited and inconsistent. A study involving Korean adolescent girls found that consuming green vegetables and fruits per day was negatively associated with depressive symptoms (14). In contrast, a study among UK adolescents did not observe significant associations between fruit and vegetable intake and depressive symptoms (15). Therefore, it is important to conduct research verifying specific dietary habits as determinants of EBPs.

Moreover, self-concept may mediate the effects of dietary habits on EBPs in children. Self-concept is a key psychological process referring to the perception and understanding of oneself (16). Previous study demonstrated that the dietary habits a person adopted could impact their self-concept. For instance, high-fat dietary intake can increase the likelihood of being overweight, leading negative self-beliefs (17). Furthermore, as suggested by Rosenberg’s the self-esteem theory, individuals tend to behave superior once they have a positive self-concept (18). In accordance with the theory, an improved self-concept resulting from healthy dietary habits may serve as a buffer against stress (19), and provide underlying motivations for coping with daily challenges (20), ultimately leading to enhanced emotional and behavioral outcomes. However, research regarding the explanation between diet and mental health has primarily focused on physiological pathways, leaving the broader range of psychological factors involved inadequately addressed.

In the present study, a range of common dietary habits will be involved to investigate their relationships with child self-concept and EBPs, expanding our understanding of the inconclusive associations of specific foods and overall healthy dietary habits with children’s mental health. Additionally, the study would innovatively explore the psychological pathways from dietary habits to EBPs via self-concept, offering a more holistic interpretive framework for the emerging field of nutritional psychiatry, which seek to understand how dietary behaviors influence mental well-being. Drawing upon these approaches, the study aimed to provide an evidence base for developing health education programs for parents, teachers, and public health policymakers to promote healthy lifestyle and positive mental development for pre-adolescents, who are in a critical period of rapid physical growth (21) and self-concept development (22). Based on previous findings, we hypothesized that a healthier diet may provide additional motivations for better emotional and behavioral outcomes through improved self-concept as shown in Supplementary Figure S1.

## 2 Methods

### 2.1 Sample

The participants were recruited for the Nutritional Knowledge, Beliefs and Behaviors Health Survey Program of Zhejiang Province, China. Stratified random sampling was used: firstly, the cities and regions in Zhejiang Province were further grouped into the economic-well-developed, economic-less-developed, and economic-undeveloped regions. Secondly, two primary schools in each economic group were randomly selected. Finally, all third-grade children and their caregivers from the above six primary schools were investigated. See Supplementary Figure S2 for the geographic distribution map.

**Figure 1:**
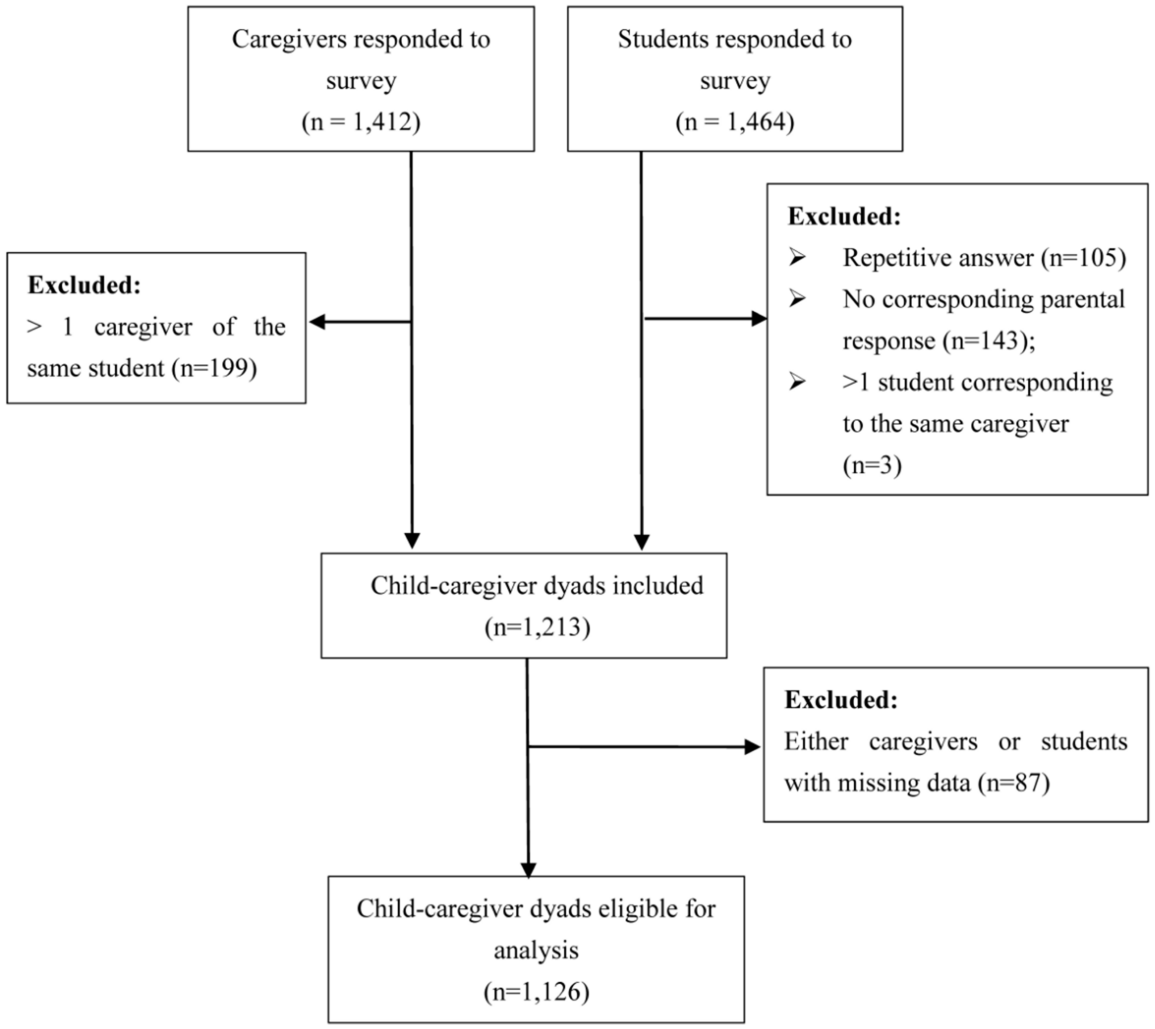
Flow chart of the inclusion and exclusion process.

Participated caregiver-child dyads with completed data were eligible for inclusion. When there was more than one caregiver for the same child, the answers from one of them were randomly selected. Details of inclusion and exclusion are displayed in Figure 1.

### 2.2 Data collection

The questionnaires used in this study were administered in electronic format. Prior to formal assessment, we organized training sessions for health investigators at each school, coordinated initial monitoring work, and offered technical support. The student surveys were administered in the school computer labs. Teachers, who had undergone prior training, guided students through the survey. Caregivers completed the survey via mobile phones. Communication platforms such as Home-School Connect and WeChat groups were used to facilitate interaction between the school and caregivers, where questions could be answered promptly.

The survey was approved by the Zhejiang Provincial Center for Disease Control and Prevention ethics review board (approval number: 2020-028-01). The written informed consent was obtained from all caregivers for themselves and their children’s participation.

### 2.3 Measures

The questionnaire in this study consists of the child reports of their dietary habits and self-concept, as well as the caregiver reports of the children’s EBPs and family demographic characteristics.

#### 2.3.1 Dietary habits

The dietary habits were evaluated through 10 questions on weekly consumption of 3 healthy foods: fresh fruit, fresh vegetables, milk/soymilk (23), as well as 7 unhealthy foods intake: sugar-sweetened beverages, fried food (23), sweet food, puffed food (24), pickled vegetables (25), Western fast food and street food (26) in the preceding week. Answers ranged from “1 day” to “7 days.” The classification and selection of foods were based on evidence-based sources derived from dietary guidelines or documented health outcomes associated with above foods (27–34).

According to the Dietary Guideline for Chinese residents (27) a daily plenty amount of vegetables, fruits, dairy products, and soybeans intake was recommended, thus having above healthy food 7 days/week was classified as a healthy level. Unhealthy food consumption ≥3 days/week was grouped into the unhealthy level based on the sample distribution and relevant literature (35). The items classified as a healthy level were assigned a score of 1; otherwise, assigned a score of 0. The final score represents an overall measure of healthy dietary habits. These items produced a Cronbach’s alpha coefficient of 0.73 in this study.

#### 2.3.2 Self-concept

The Piers-Harris Children’s Self-Concept Scale (PHCSS) is an 80-item self-reported scale designed for evaluating a child’s self-concept in 6 domains: Intellectual and School Status (ISS), Physical Appearance and Attributes (PAA), Freedom from Anxiety (FA), Popularity (PPL), Happiness and Satisfaction (HS), and Behavioral Adjustment (BA). Children were required to answer “yes” (1) or “no” (0) to each item, with a total score of 80. A score below the 30th percentile (corresponding to a score of 46) indicates a lower self-concept based on the norms established for Chinese children by Huang et al. (36) and Linyan et al. (37). The Cronbach’s alpha coefficients of the Chinese version of PHCSS was 0.86 among 8- to 16 year-old-children (36, 37).

#### 2.3.3 Emotional and behavioral problems

The Rutter’s Child Behavior Questionnaire (RCBQ)-Parent Version is a scale to detect children’s EBPs at home or school during the last 12 months (38). It is a 31-item scale with each item rated from 0- never, 1- occasionally/less than once a week, to 2- occurred at least once a week, with a total score ranged from 0 to 62. A total score of 13 as the cut-off point was used to identify children with deviant behavioral and emotional status with specificity of 94% and sensitivity of 74% among Chinese children (38). The reliability test for the Chinese version yielded Cronbach’s alpha coefficients ranged from 0.63 to 0.80 (39, 40).

The RCBQ was classified into three subscales based on Rutter’s symptomatology interviews: (i) neurotic behaviors that represent different aspects of emotional difficulties; (ii) anti-social behaviors that estimate the conduct problems (iii) mixed behaviors containing the rest of problematic behaviors, such as hyperactive (41). Wong (38) further performed the factor discriminant analysis, showing three subscales had correct classification rates of 90% for conduct problems and 76% for emotional problems among Chinese children.

#### 2.3.4 Covariates

Demographic characteristics were also obtained from caregivers, including child age, residential district (urban, rural–urban fringe, and rural), respondents (father, mother, grandparents, and other), existence of siblings (yes, no), maternal and parental education (three levels), and family annual per capita income (three levels, from <¥50,000 to >¥100,000).

### 2.4 Statistics analysis

To describe the general characteristics of participants and corresponding EBPs scores, the number (*N*) and percentage (%) of included parent–child dyads were presented.

Multilevel logistic regression analysis was employed to assess the odds ratios (OR) and 95% confidence intervals (CIs) for the associations of dietary habits with self-concept and EBPs in children accounting for variance at different income levels. Given that all the data of dietary habits and self-concept were both obtained from the child self-reports, the common method bias was examined using Harman’s single factor test. According to the recommended threshold, the presence of a single factor accounting for more than 40% of the total variance indicates significant common method bias (42).

As normality test of above variables showed Skewness ranged from −0.84 to 1.01 and Kurtosis ranged from −0.23 to 1.09, indicating no significant deviation (Skewness <|2.0| and Kurtosis <|7.0|) in continuous variables (43). Therefore, the Pearson correlation coefficient (*r*) was used to examine the correlations among continuous variables (overall healthy dietary habits, self-concept and EBPs).

Mediation analysis was performed to examine the direct and indirect effects of healthy dietary habits and self-concept on EBPs. The mediation effect was assessed using Baron and Kenny’s multiple regression approach, with the mediation estimates computed via the product of path coefficients (44). Covariates were all adjusted in the model. The hypothesized relationships were quantified by standardized regression coefficients (*β*) and standard error (SE), using a maximum likelihood estimator with 5,000 bootstrapped samples.

Analyses were run with no missing data. The SPSS 25.0 (IBM Corporation, Armonk, NY, United States) (45) was used for the data organization and descriptive analyses. Correlation and mediation analyses were conducted by the mediation package (v.4.5.0) in R 4.0.3 (R Core Team, 2020) (46). All *p*-values in this study were two-tailed, with *p* < 0.05 considered statistically significant.

## 3 Results

### 3.1 Characteristics of the studied population

**Figure 2:**
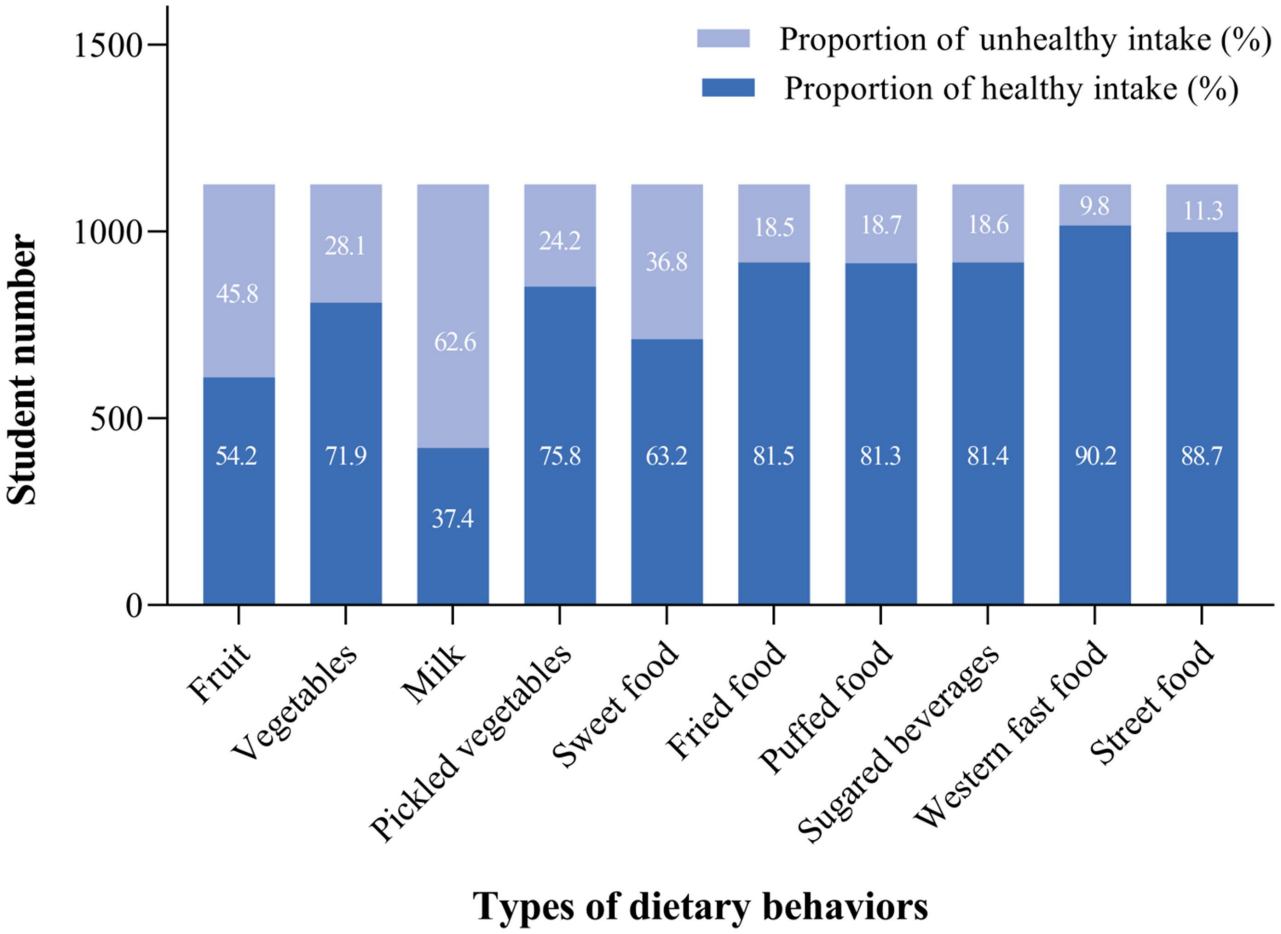
Proportion of children with healthy dietary habits.

One thousand one hundred and twenty-six caregiver-child dyads were included in the study. Among included children (47.2% girls), the mean age was 9.53 ± 0.66 years, with slightly higher percentages of urban residents (71.3%) and mother respondents (67.1%). Using the established cut-off score of scales, 216 children (19.1%) were identified as having a low self-concept, and 302 children (26.8%) exhibited parent-reported deviant EBPs (Table 1). As shown in Figure 2, only 37.4 and 54.2% of children met the healthy level of milk/soy milk and fruit intake respectively, and the healthy rate of sweet food (63.2%), vegetable (71.9%), and pickled vegetable (75.8%) intake was also relatively low compared to other food groups.

**Table 1:** Characteristics of participated family by child EBPs levels (*n* = 1,126).

Variables	Total sample	EBPs Level		*p*-value
		Deviated *n* = 302	Normal *n* = 824	
Age (M ± SD)	9.53 ± 0.66	9.52 ± 0.67	9.53 ± 0.66	0.916
Sex, *n* (%)				0.001*
Boys	595 (52.8)	184 (60.9)	411 (49.9)	
Girls	531 (47.2)	118 (39.1)	413 (50.1)	
District, *n* (%)				0.367
Urban	803 (71.3)	209 (69.2)	594 (72.1)	
Rural–urban fringe	188 (16.7)	50 (26.6)	138 (16.7)	
Rural	135 (12.0)	43 (14.2)	92 (11.2)	
Caregiver respondent, *n* (%)				0.420
Father	260 (23.1)	70 (23.2)	190 (23.0)	
Mother	755 (67.1)	208 (68.9)	547 (66.4)	
Grandparents and other	111 (9.8)	24 (7.9)	87 (10.6)	
Existence of siblings, *n* (%)				0.006*
Yes	744 (66.1)	299 (78.3)	525 (70.6)	
No	382 (33.9)	83 (21.6)	219 (29.4)	
Maternal education level, *n* (%)				0.522
Primary education or illiterate	127 (11.3)	33 (10.9)	94 (11.4)	
Secondary education	582 (51.7)	149 (49.3)	433 (52.5)	
Tertiary education	417 (37.0)	120 (39.7)	297 (36.0)	
Paternal education level, *n* (%)				0.682
Primary education or illiterate	119 (10.6)	32 (10.6)	87 (10.6)	
Secondary education	608 (54.0)	157 (52.0)	451 (54.7)	
Tertiary education	399 (35.4)	113 (37.4)	286 (34.7)	
Annual per capita income, *n* (%)				0.275
<50,000 ¥	321 (28.5)	88 (29.1)	233 (28.3)	
50,000–100,000 ¥	369 (32.8)	108 (35.8)	261 (31.7)	
>100,000 ¥	436 (38.7)	106 (35.1)	330 (40.0)	
Self-concept levels, *n* (%)				<0.001*
Low (≤45)	215 (19.1)	83 (27.5)	132 (16.0)	
Normal (>45)	911 (80.9)	219 (72.5)	692 (84.0)	

EBPs, emotional and behavioral problems. Values were presented as number (*n*) with percent (%). *p < 0.05, statistical significant.

### 3.2 Different dietary habits associated with self-concept and EBPs

The results of Harman’s single-factor test revealed four factors with eigenvalues exceeding 1. The first factor accounted for 24.03% of the total variance, which is below the established threshold of 40% (42). The findings suggest that common method bias was not a significant concern in this study.

**Figure 3:**
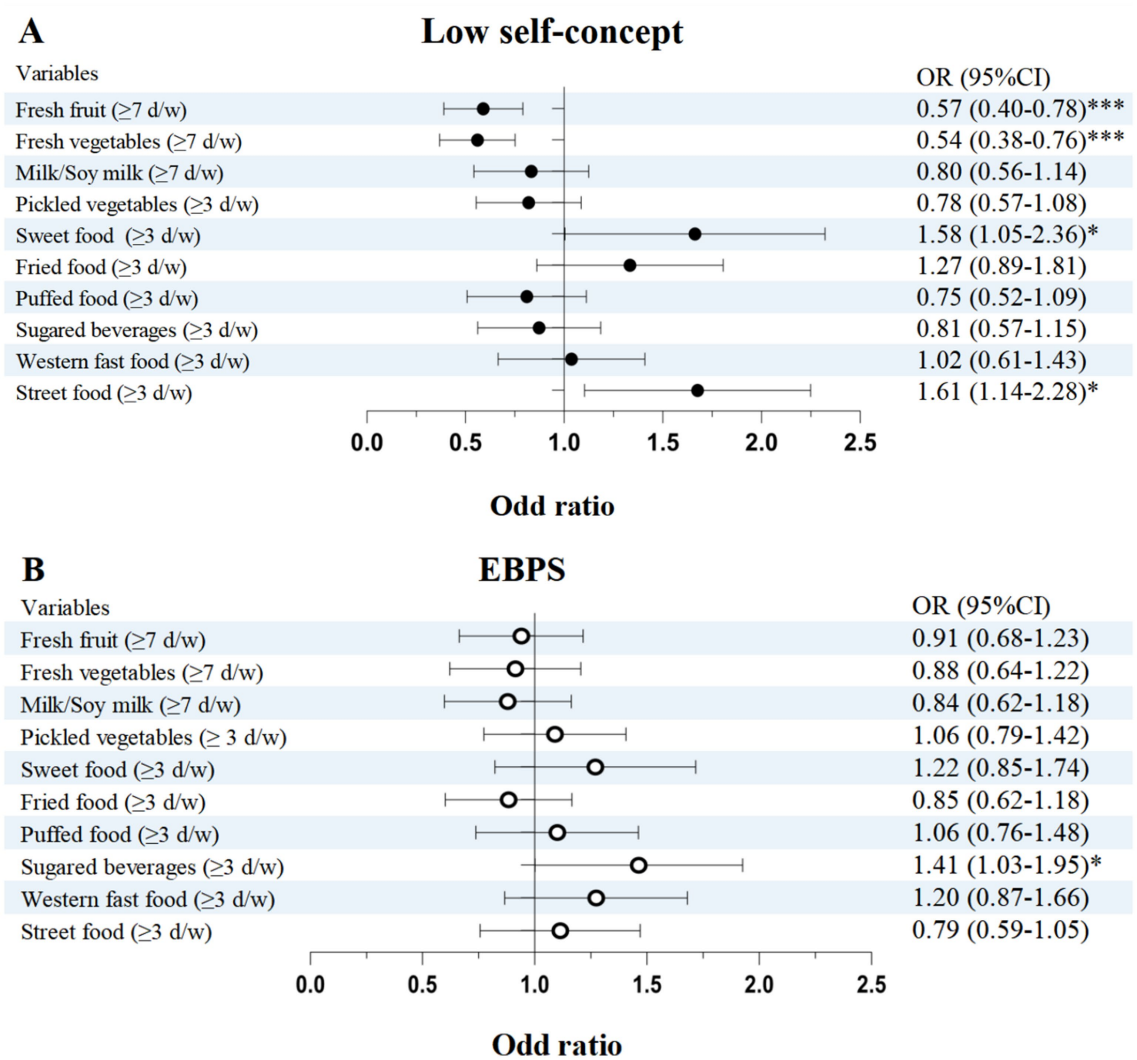
Multilevel logistic regression analysis of dietary habits associated with self-concept **(A)** and EBPs **(B)**. ^*^p < 0.05, ^**^p < 0.01, ^***^p < 0.001. CI, confidence interval; EBPs, emotional and behavioral problems; d, day; w, week. Model was adjusted for child age, sex, residence, caregiver respondents, parental educational level, and existence of sibling.

In Figure 3, the results of multilevel logistic regression model adjusted for all covariates showed that, compared with the unhealthy dietary intake, the healthy fresh fruit (OR = 0.57, 95% CI 0.40–0.78, *p* = 0.001) and vegetables intake (OR = 0.54, 95% CI 0.38–0.76, *p* < 0.001) had negative associations with low self-concept. Having sweet foods (OR = 1.58, 95% CI 1.05–2.36, *p* = 0.037) and street foods (OR = 1.61, 95% CI 1.14–2.28, *p* = 0.026) over three times a week were associated with higher likelihood of low self-concept. In the model of EBPs, the unhealthy sugar-sweetened beverages (OR = 1.41, 95% CI 1.03–1.95, *p* = 0.034) was associated with increased risk of EBPs.

### 3.3 Correlation among healthy dietary habits, self-concept and EBPs

Table 2 presents the bivariate associations result among continuous variables. Healthy dietary habits were positively correlated with total self-concept scores (*r* = 0.29, *p* < 0.001) and all dimensions of self-concept (*r* = 0.15–0.26, *p* < 0.001). Meanwhile, negative correlations were seen between healthy dietary habits and overall EBPs (*r* = −0.13, *p* < 0.001). In detailed aspects of EBPs, anti-social behaviors (*r* = −0.14, *p* < 0.001) and mixed behaviors (*r* = −0.13, *p* < 0.001) were correlated with healthy dietary habits, whereas the association between healthy dietary habits and neurotic behaviors was insignificant (*r* = 0.05, *p* = 0.106).

**Table 2:** Bivariate correlations among overall healthy dietary habits, self-concept and EBPs.

Variables	M ± SD	1	2	3	4	5	6	7	8	9	10	11	12
1. Healthy dietary habits	7.26 ± 2.02	/											
2. Self-concept	56.10 ± 11.41	0.29^***^	/										
3. ISS	11.21 ± 3.37	0.24^***^	0.82^***^	/									
4. PAA	8.05 ± 3.02	0.18^***^	0.73^***^	0.69^***^	/								
5. FA	8.96 ± 2.79	0.19^***^	0.73^***^	0.51^***^	0.36^***^	/							
6. PPL	8.51 ± 2.18	0.24^***^	0.78^***^	0.55^***^	0.49^***^	0.64^***^	/						
7. HS	7.50 ± 1.74	0.15^***^	0.74^***^	0.51^***^	0.59^***^	0.54^***^	0.54^***^	/					
8. BA	12.80 ± 2.67	0.26^***^	0.81^***^	0.60^***^	0.42^***^	0.55^***^	0.59^***^	0.53^***^	/				
9. EBPs	10.33 ± 5.85	−0.13^***^	−0.18^***^	−0.12^***^	−0.08^*^	−0.15^***^	−0.15^***^	−0.11^***^	−0.19^***^	/			
10. Neurotic	1.10 ± 1.48	−0.05	−0.16^***^	−0.09^**^	−0.10^**^	−0.17^***^	−0.13^***^	−0.13^***^	−0.14^***^	0.76^***^	/		
11. Anti-social	0.93 ± 1.21	−0.14^***^	−0.11^***^	−0.10^***^	−0.02	−0.06	−0.10^**^	−0.05	−0.18^***^	0.76^***^	0.46^***^	/	
12. Mixed	8.50 ± 4.10	−0.13^***^	−0.10^**^	−0.11^***^	−0.07^*^	−0.15^***^	−0.08^*^	−0.10^**^	−0.11^***^	0.97^***^	0.59^***^	0.52^***^	/

^*^p < 0.05, ^**^p < 0.01, ^***^p < 0.001. EBPs, emotional and behavioral problems; ISS, intellectual and school status; PAA, physical appearance and attributes; FA, freedom from anxiety; PPL, popularity; HS: happiness and satisfaction; BA, behavioral adjustment.

A significant inverse association was also observed between total self-concept and total EBPs (*r* = −0.18, *p* < 0.001). Concerning the links among different dimensions of self-concept and EBPs, results showed that anti-social behaviors were not related to the self-concept with respect to physical appearance and attributes (*r* = −0.02, *p* = 0.418), freedom from anxiety (*r* = −0.06, *p* = 0.06), and happiness and satisfaction (*r* = −0.05, *p* = 0.09), while other aspects of self-concept and EBPs were all negatively correlated with each other (*r* ranged from −0.07 to −0.18, *p* all <0.02).

### 3.4 Mediating role of self-concept in association between healthy dietary habits and EBPs

**Figure 4:**
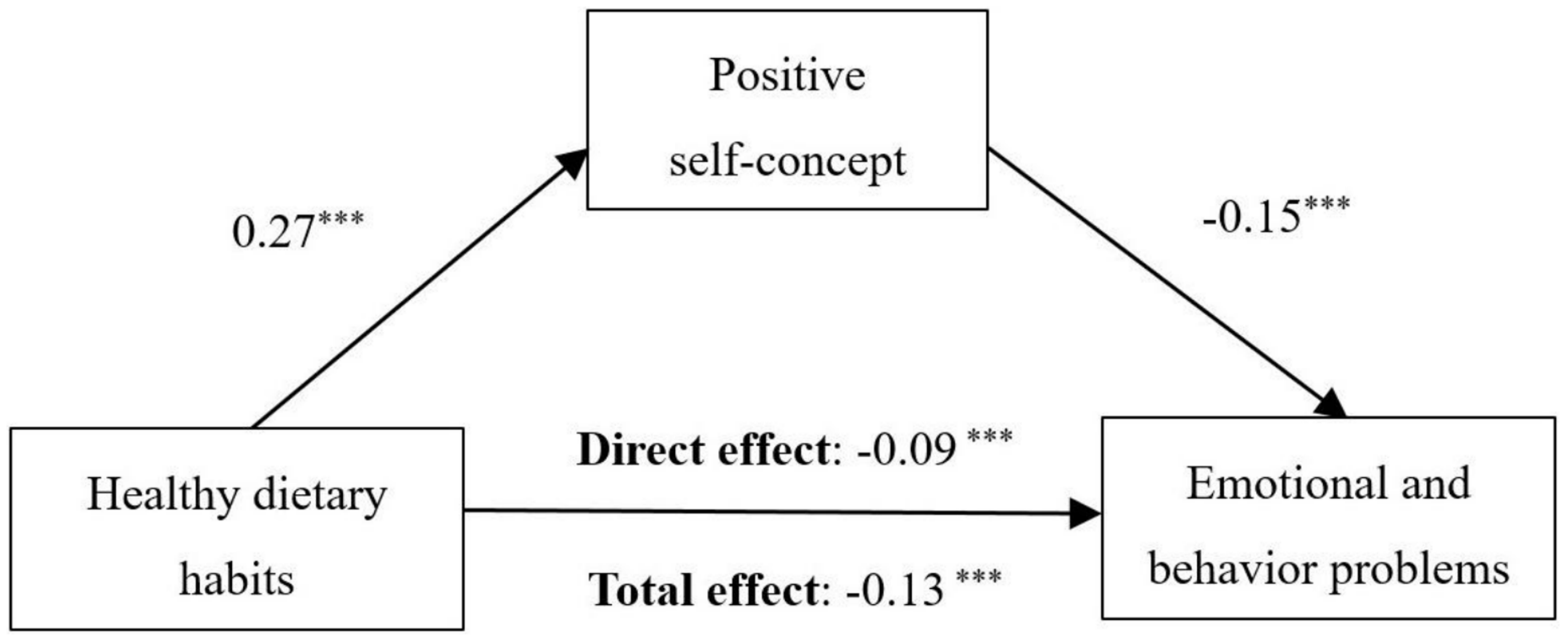
Mediation analysis of the role of self-concept in the relationship between dietary habits and emotional and behavioral problems. ^*^p < 0.05, ^**^p < 0.01, ^***^p < 0.001. EBPs, emotional and behavioral problems. Model was adjusted for child age, sex, district, caregiver respondent, existence of sibling, parental educational levels, and family annual per capita income. Standardized regression coefficients *β* are displayed.

Based on the hypothesized model and bivariate correlation analysis, examination of direct effects on children’s EBPs showed that healthy dietary habits (*β* = −0.09, SE *=* 0.09, *p =* 0.003) and better self-concept (*β =* −0.15, SE *=* 0.02, *p <* 0.001) were associated with lower levels of children’s EBPs. Results of indirect effect revealed that self-concept partially mediated the relationship between children’s dietary habits and EBPs with an indirect effect of *β =* −0.04, 95%CI −0.06 to −0.02, *p* < 0.001, which led to a total effect of healthy dietary habits on EBPs, *β =* −0.13, 95%CI −0.19 to −0.07, *p* < 0.001, the proportion of mediation was 29% (see Figure 4 and Table 3).

**Table 3:** Mediation analysis of relationship between healthy dietary habits, self-concept and EBPs.

	Standardized effect	Bootstrap 95% CIs		*p*
		Lower	Upper	
Direct effect	−0.09	−0.15	−0.03	<0.001
Indirect effect	−0.04	−0.06	−0.02	<0.001
Total effect	−0.13	−0.19	−0.07	<0.001
Proportional mediated	0.29	0.14	0.58	<0.001

Model was adjusted for child age, sex, family income, parental educational level, respondents and existence of siblings. EBPs, emotional and behavioral problems.

## 4 Discussion

In this school-based study of 1,126 Chinese parent–child dyads, we found children’s frequent sugar-sweetened beverages was linked to more EBPs. Unhealthy intake habits of sweets and street food are associated with a negative self-concept. However, children who had a healthy intake of fruit and vegetable were less likely to have a low self-concept. Furthermore, we found self-concept may explain the association between healthy dietary habits and EBPs.

In this study, we conducted a food frequency evaluation of representative third-grade students in the Zhejiang province of China. Notably, we found a relatively low proportion of healthy milk or soy milk (37.4%) and fruit (54.2%) intake, as well as a high proportion of unhealthy sweet food (36.8%) intake compared to other food items. These results are comparable to a similarly low 48.8% healthy rate of fruit intake in nationwide students aged 8–9 years in Italy (47), and lower than the unhealthy rate of milk intake in European (48) and Japanese children using the same cut-off (49). It is also worth mentioning a relatively low prevalence of Western fast food intake among children in China compared to those in Western countries (50, 51). To add to the previous knowledge, our study also revealed the unhealthy rate of Eastern-style dietary habits such as frequent intake of pickled vegetables (50.1%) and street food (4.4%) among children, which has shown related to negative health consequences (52, 53).

While comprehensively examining the linkage between different food intakes and EBPs, our study revealed that children with low sugar-sweetened beverages had decreased odds of EBPs compared with children with high intake. It is aligned with several prior findings. For instance, a meta-analysis of observational studies demonstrated that sugared-sweetened beverages were positively related to the risks of individuals’ ADHD symptoms (pooled OR = 1.22, 95%CI: 1.04–1.42) (54). Meanwhile, our finding also aligns with a population-based study in Chinese children and adolescents, that suggested more frequent consumption of sugar-sweetened beverages was related to higher risk of EPBs (55). The main hypothesized mechanism of such association is the higher glycemic index induced by sugar-sweetened beverages relative to other foods (56). The increased insulin released and brain serotonin concentration after sugary soft drinks are closely linked to emotional dysregulation and later distress (57). Another study also highlighted the negative effects of sodium benzoate, a substance typically contained in beverages. It showed that sodium benzoate may decrease glutathione but elevate malondialdehyde levels in the brain, which subsequently leads to behavioral problems in children (58). Nevertheless, it should be cautious that, the EBPs development cannot be simply ascribed to specific food intake and its physiological alteration, due to the food synergy effect (59) and complex etiological factors of EBPs (60).

Numerous prior studies have also focused on the relationship between vegetable and fruit intake and child EBPs. Our findings agree with Winpenny et al. (15) that suggesting no association between fruit and vegetable intake (serving/day) and child depressive symptoms in UK. Yet, previous studies also showed healthy fruit and vegetable intake <once/day linked to fewer inattention problems (61), and fruit and more vegetable and fruit diet component was associated with less EBPs in children (62). However, previous studies exhibited the discrepancies in measuring food intake and children’s EBPs, which may be the main source of differences in results. Therefore, future research with more uniformity in the instruments and prospective design is warranted to solve the indefinite conclusion. Lastly, regarding overall dietary habits, our finding of no direct correlation between overall healthy diet and EBPs somewhat contradicted previous studies. For example, the meta-analysis of Orlando for 39 observational studies revealed a significant association between a healthy diet and emotional problems in individuals ≤18 years old (63). This conflicting result may be partially attributed to the relatively lower prevalence of unhealthy dietary intake included in this study, such as fried foods and Western fast foods, among children in China compared to those in Western countries that were predominantly included in previous research. Future research should conduct more cross-population studies to examine the role of specific ethnicity, dietary cultures in the linkage between dietary habits and mental health problems.

The study also demonstrated an association between overall healthy dietary habits and self-concept. This finding validated the previous findings indicating that adherence to the Mediterranean diet, which is a typically recognized healthy dietary pattern characterized by fruits, vegetables, and oily fish, is associated with self-concept in students from different countries (64, 65). In terms of different foods intake and self-concept, our results indicated the healthy intake of fresh fruit and vegetables was the protective factor for low self-concept, while the unhealthy consumption of sweet foods was detrimental. These findings contribute to the existing evidence by expanding our understanding of the relationship between various food components and child self-concept, which can be explained in the following two aspects. Physically speaking, dietary intake of healthy foods is important to maintain physical fitness. For example, sufficient legumes as a kind of vegetable that contains proteins and fiber can help improve glycemic control and reduce blood lipids (66). By contrast, significant number of calories from sweets or snacks (67), as well as substantial amounts of fat, trans-fat and salt, brought by street food are all closely linked to obesity and cardiovascular risks (52). As such, children’s weight and shape may be impacted differently from unhealthy and unhealthy foods intake which ultimately contributes to a different body image that boosts or ruins their physical self-concept (17). Psychologically speaking, good dietary habits have been found helpful to learning and attention through modulation of neurotransmitters (68) or improve cerebral blood flow and oxygenation (69) which are correlated to higher academic performance and better cognition self-concept (70). Therefore, the investigation of mediated nutritional biomarkers is the important next step to an in-depth understanding of the association between food intake habits and child psychological well-being.

Furthermore, the exploration of psychophysiological pathways indicated that children’s self-concept partially mediated the positive relationship between a healthy diet and fewer EBPs. This is consistent with the self-esteem theory, which posits that individuals with positive self-concept are more likely to engage in positive behaviors (18). In light of this, it can be argued that for children and adolescents, an improved self-concept, which may results from a healthy diet, can help people sustain a stable sense of self and achieve emotionally stability due to less perceived negative feedback or their usage of other important aspects of themselves to counteract such feedback (71). As a result, they are more motivated to set and achieve goals, take on challenging tasks, and exhibit less EBPs that reinforce their positive self-image (18, 20). For instance, those children with adherence to a healthy daily diet usually with less weight-related problems and positive physical self-concept (72, 73) than those can protect them from EBPs by buffering them against the self-dissatisfaction derived from fat talk and weight-related bullying (23). The findings of our study provide implications in future health education that developing healthy eating habits is crucial for the mental health of children. Efforts should be made toward promoting awareness of healthy eating habits in children, especially increasing fruit and vegetable consumption and reducing intake of sugar-sweetened beverages and sweet food, possibly via offering sufficient nutritious food options, educating caregivers about balanced meals, and incorporating nutrition education into school curricula. By forming healthy eating habits at home and school, children can develop lifelong habits that positively impact their self-concept and mental health.

## 5 Strengths and limitations

This epidemiological study among pre-adolescents provides valuable insights into the current state of their dietary habits, the critical dietary factors associated with mental health, and the underlying mechanisms mediating these relationships. Moreover, it is the first study to examine associations between various dietary factors and self-concept, as well as the potential psychological pathway linking diet to EBPs. It added knowledge of different healthy and unhealthy food intake related to self-concept and EBPs to a previously limited evidence base. Moreover, the present study provides empirical evidence of self-esteem theory by suggesting that the enhanced self-concept derived from a healthy diet may also contribute to better mental health in children. Lastly, by presenting the current status of child dietary habits and their influential pathways to EPBs, it helps local governments and educational institutions formulate culturally appropriate nutritional improvement policies, which further support the United nations’ sustainable goals that improve the quality and accessibility of nutritious food options. Furthermore, the study provides a roadmap for the further nutritional education for an integrated health service to children, which helps create a supportive environment for children to thrive, both physically and mentally.

Our study is also subject to several limitations. Firstly, due to the cross-sectional design of the study, we were unable to establish a causal relationship between diet intake and changes in EBPs over time. Therefore, prospective studies are needed to further clarify the temporal sequence of dietary habits and EBPs, thereby strengthening causal inferences. Secondly, self-reported measures were thoroughly employed during the assessment, while efforts were made to provide survey instructions, employ of reliable and clear scales, as well as demonstrate the absence of significant common method bias, it nevertheless introduced potential limitations to the validity of the findings in three key respects: firstly, the dietary habits survey relies on children’s retrospective reports of their weekly eating habits but daily records, potentially introducing recall bias. This is especially relevant for children, who often follow meal plans set by others, making some eating habits intuitive. Furthermore, using online electronic questionnaires instead of offline interviews makes it difficult to fully ensure the quality of completion. Especially for pre-adolescents whose sustained attention is easy to be challenged by the large number of questions, potentially affecting the accuracy of their self-reported responses. Thirdly, parents may be difficult to detect child behavioral problems outside their presence or subtle child emotional problems, leading to underreporting of certain problems. Future research is encouraged to utilize 24-h dietary recalls and multiple-informant assessments of child EBPs, to implement one-on-one interviews, as well as to incorporate conditional questions during the data cleaning, which can aid in the accuracy of self-reported results. Lastly, regarding the generalizability, it is important to note that our study only focused on families in Zhejiang, an economically developed province in eastern China, therefore, it should be cautious to extrapolate the results to different populations as the regional variations in socio-economic status could influence the observed associations. Future surveys should be carried out in different regions of China, and even globally, so as to formulate nutritional supply plans that are tailored to various local conditions.

## 6 Conclusion

In this investigation of third-grade children and their caregivers, this study reveals a low proportion of children had the healthy foods intake of milk and fruits. The study also demonstrated a higher intake of fresh fruits and vegetables was linked to a higher self-concept, while frequent consumption of sweet and street foods was associated with a lower self-concept, unhealthy sugar-sweetened beverage intake was found to elevate the risk of EBPs. Furthermore, as an underlying psychological pathway, a healthy diet may help improve children’s self-concept and finally benefit their mental health. The study has not only validated the association between diet and mental health but also elucidated the psychosocial mechanisms underlying this relationship. Future policies should be planned for a healthy diet in children and the cultivation of healthy eating habits should be provided as support for children’s mental health and well-being.

## Data Availability

The raw data supporting the conclusions of this article will be made available by the authors, without undue reservation.
