# Supplementary material for: Association between dietary habits and emotional and behavioral problems in children: the mediating role of self-concept
Source: Front Nutr. 2025 Mar 7;12:1426485. doi: 10.3389/fnut.2025.1426485 (PMC11925766; doi:10.3389/fnut.2025.1426485)
Supplement: Supplementary file 1 [file Image_1.pdf]

Supplementary Material

1 Supplementary Figures

Figure S1. The hypothesized self-enhancement pathways of child emotional and behavioral problems

Figure S2. The geographic location of participants of the Nutritional Knowledge, Beliefs and Behaviors Health Survey

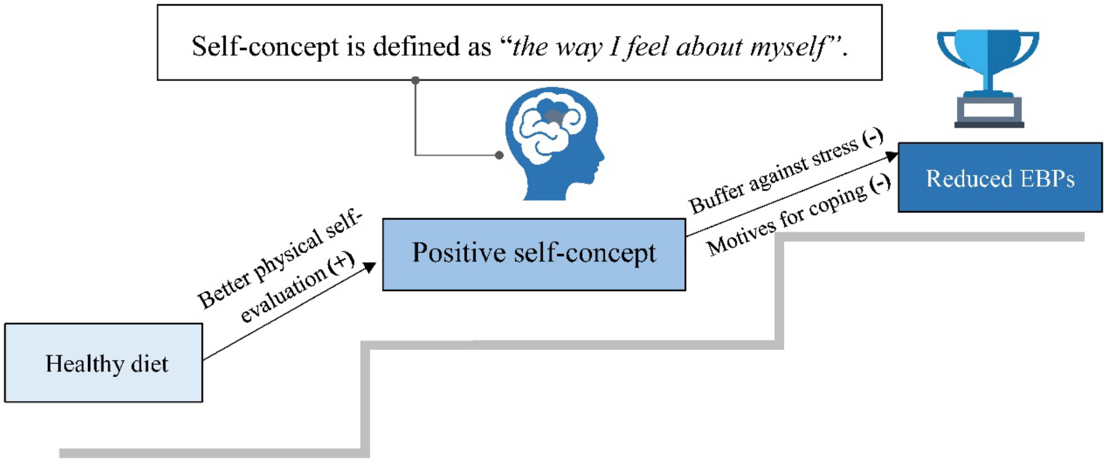

Figure S1. The hypothesized self-enhancement pathways of child emotional and behavioral problems

Note: EBPs, Emotional and behavioral problems.

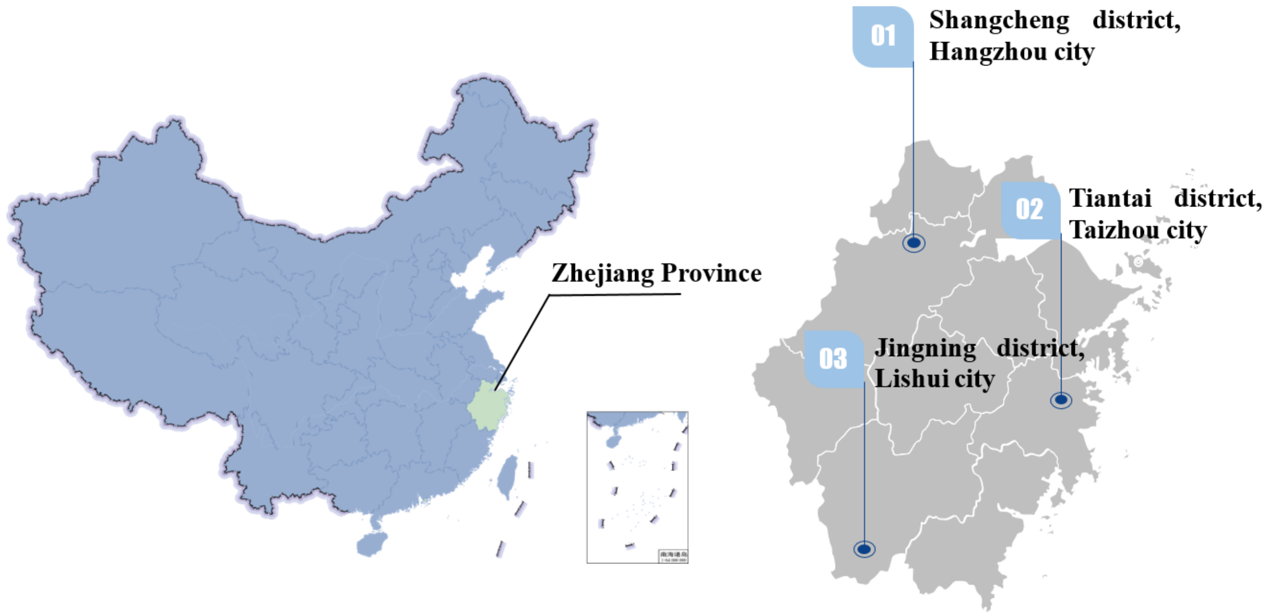

Figure S2. The geographic location of participants of the Nutritional Knowledge, Beliefs and Behaviors Health Survey
